# Propionyl Carnitine Metabolic Profile: Optimizing the Newborn Screening Strategy Through Customized Cut-Offs

**DOI:** 10.3390/metabo15050308

**Published:** 2025-05-06

**Authors:** Maria Lucia Tommolini, Maria Concetta Cufaro, Silvia Valentinuzzi, Ilaria Cicalini, Mirco Zucchelli, Alberto Frisco, Simonetta Simonetti, Michela Perrone Donnorso, Sara Moccia, Ines Bucci, Maurizio Aricò, Vincenzo De Laurenzi, Luca Federici, Damiana Pieragostino, Claudia Rossi

**Affiliations:** 1 Center for Advanced Studies and Technology (CAST), “G. d’Annunzio” University of Chieti-Pescara, 66100 Chieti, Italy; maria.tommolini@unich.it (M.L.T.); maria.cufaro@unich.it (M.C.C.); silvia.valentinuzzi@unich.it (S.V.); ilaria.cicalini@unich.it (I.C.); mirco.zucchelli@unich.it (M.Z.); alberto.frisco@unidav.it (A.F.); ines.bucci@unich.it (I.B.); vincenzo.delaurenzi@unich.it (V.D.L.); luca.federici@unich.it (L.F.); damiana.pieragostino@unich.it (D.P.); 2 Department of Innovative Technologies in Medicine and Dentistry, “G. d’Annunzio” University of Chieti-Pescara, 66100 Chieti, Italy; sara.moccia@unich.it; 3 Department of Science, “G. d’Annunzio” University of Chieti-Pescara, 66100 Chieti, Italy; 4 Department of Human, Legal and Economic Sciences, Telematic University of “Leonardo Da Vinci”, 66010 Torrevecchia Teatina, Italy; 5 Regional Centre for Neonatal Screening, Department of Clinical Pathology and Neonatal Screening, Children’s Hospital “Giovanni XXIII”, Azienda Ospedaliero-Universitaria Consorziale, 70126 Bari, Italy; simonetta.simonetti@policlinico.ba.it; 6 Laboratory for the Study of Inborn Errors of Metabolism, Pediatric Clinic, IRCCS Instituto Giannina Gaslini, 16147 Genoa, Italy; michelaperronedonnorso@gaslini.org; 7 Department of Neuroscience, Rehabilitation, Ophthalmology, Genetics, Maternal and Child Health, University of Genoa, 16126 Genoa, Italy; 8 Department of Medicine and Aging Science, “G. d’Annunzio” University of Chieti-Pescara, 66100 Chieti, Italy; 9 U.O.C. Pediatrics, S. Spirito Hospital, ASL Pescara, 65124 Pescara, Italy; maurizio.arico@asl.pe.it

**Keywords:** expanded newborn screening, tandem mass spectrometry, inborn errors of metabolism, second-tier test, false positive, postanalytical interpretative tools

## Abstract

Background: The advent of tandem mass spectrometry (MS/MS) had an essential role in the expansion of newborn screening (NBS) for different inborn errors of metabolism (IEMs). Nowadays, almost 50 IEMs are screened in Italy. The use of second-tier tests (2-TTs) in NBS minimizes the false positive rate; nevertheless, the metabolic profile is influenced not only by the genome but also by environmental factors and clinical variables. We reviewed the MS/MS NBS data from over 37,000 newborns (of which 8% required 2-TTs) screened in the Italian Abruzzo region to evaluate the impact of neonatal and maternal variables on propionate-related primary biomarker levels. Methods: Expanded NBS and 2-TT analyses were performed using MS/MS and liquid chromatography–MS/MS methods. We set up layered cut-offs dividing all 37,000 newborns into categories. Statistical analysis was used to create alarm thresholds for NBS-positive samples. Statistically significant differences were found in both neonatal and maternal conditions based on the 2-TTs carried out. According to the stratified cut-offs, only 1.47% of the newborns would have required a 2-TT while still retaining the ability to recognize the true-positive case of methylmalonic acidemia with homocystinuria, which has been identified by NBS. To further support the clinical applicability, we performed an external evaluation considering nine positive cases from an extra-regional neonatal population, confirming the potential of our model. Interestingly, the setting of alarm thresholds and their application would allow for establishing the degree of priority/urgency for 2-TTs. Conclusions: Tailoring NBS by customized cut-offs may enhance the application of precision medicine, focusing on true-positive cases and also reducing analysis costs and times.

## 1. Introduction

Newborn screening (NBS) is a public health preventive medicine program aimed at supporting the early diagnosis of different inborn errors of metabolism (IEMs), which, if not promptly identified and treated during the presymptomatic phase, within the first days of life, could compromise the child’s health and survival [1]. The advent of tandem mass spectrometry (MS/MS) in the 1990s allowed for an impressive expansion of NBS, a crucial breakthrough in many screening programs. The multiplexing approach in the expanded NBS led to the new concept of “*one test for plenty of diseases*” through metabolic profiling by MS/MS on a single dried blood spot (DBS), overcoming the traditional NBS strategy based on “*one test for one disease*” [2]. In Italy, the national law 167/2016 extended the mandatory NBS to almost 50 IEMs and established the Coordination Center for NBS at the “Istituto Superiore di Sanità” (ISS) to ensure homogeneity throughout the regional NBS programs. To date, the Italian expanded NBS program includes congenital hypothyroidism, cystic fibrosis, aminoacidopathies, including phenylketonuria (PKU) [3], urea cycle disorders [4], organic acidurias [5], beta oxidation fatty acid deficiencies [6], galactosemia [7], and biotinidase deficiency [8,9]. NBS for IEMs relies on the measurement of acylcarnitines and amino acids in dried blood spot samples by MS/MS. NBS tests are not diagnostic; however, abnormal biomarker levels are used to assess the risk of a disorder in asymptomatic newborns. The major challenge of every screening program is to avoid false negatives and also to minimize the false positive rate, thus reducing the number of newborns requiring confirmatory tests [2,10,11,12]. This is accomplished by selecting appropriate cut-offs and algorithms, aiming to obtain maximum sensitivity and specificity. Nevertheless, for some disorders, due to genomic variants, environmental influences, medications, and several additional maternal and neonatal factors [5,13,14], a high number of false-positive results can be expected.

In this context, the use of biomarker ratios and the application of second-tier test (2-TT) strategies can minimize the number of false positives, thus avoiding unnecessary recalls, since 2-TTs are performed on the same sample used for the primary screening [15]. This approach is carried out to quantify either the target analyte of the first tier or additional diagnostic markers, thus gaining higher specificity and improving the interpretation of abnormal results at NBS. Therefore, the positive predictive value (PPV) is increased accordingly [11].

In our NBS lab experience, the highest false-positive results are related to tests targeting inborn errors of propionate, cobalamin, and methionine metabolism; therefore, we apply a 2-TT for the differential diagnosis of methylmalonic aciduria—MMA, propionic aciduria—PA, homocystinuria, and remethylation disorders through the simultaneous quantification of methylmalonic acid (mma), methylcitric acid (mca), and homocysteine (hcy) from DBS [1]. Precisely, this 2-TT strategy is performed when an alteration of propionyl-carnitine (C3) or methionine levels occurs. The isolated elevation of C3 levels alone is not a specific marker for MMA and PA [5], and it is associated with high FPRs, even if coupled to specific analyte ratios and to the new biomarker heptadecanoylcarnitine C17 (C16:1-OH isobar) [11,15,16]. So far, in our experience, the percentage of newborns requiring this 2-TT is about 8% [5,11,15]. The 2-TT strategy reduces the need to recall newborns at the price of a significant increase in the laboratory workload. Therefore, it is worth trying to improve the specificity of the primary marker by taking into account all those preanalytical factors that can influence its values. Indeed, the overall efficiency of the NBS workflow can be improved with postanalytical interpretative tools, which allow for adjusting NBS data based on clinical variables, including birth weight (BW), gestational age (GA), and time (age or hours) at blood collection, as well as antibiotic and/or cortisone treatment and type of nutrition, which could contribute to increasing the false-positive rate and the necessity of 2-TTs [17].

In order to evaluate the impact of preanalytical variables on propionate metabolism and to improve the interpretation of the results from the first- and second-level tests at NBS, we reviewed the MS/MS NBS data of 37,000 newborns screened in the Abruzzo region from February 2019 to June 2023. Of them, 2987 required a 2-TT due to alterations in propionate metabolism markers at the primary screening test. Thirty-four infants tested positive at the 2-TT, seven were referred for diagnostic confirmation, and only one ended up being diagnosed with MMA with homocystinuria. The discrepancy between the high percentage of 2-TTs performed and a single true-positive case prompted us to calculate new cut-offs adjusted for BW, GA, and age at sampling to refine the NBS workflow by improving the specificity of the primary biomarker, thus reducing the number of 2-TTs. Moreover, we analyzed the effect of neonatal and maternal variables on the propionate-related markers to obtain information useful to define 2-TT priority.

## 2. Materials and Methods

### 2.1. Newborn Screening Analysis and Second-Tier Testing

Expanded NBS and 2-TT analysis on DBS were performed following the FIA-MS/MS and LC-MS/MS methods, as previously described by Rossi et al. [5]. Briefly, for routine NBS, 3.2 mm disks of DBS samples were extracted for the determination of 14 AAs, 35 acylcarnitines (ACs), free carnitine, and succinylacetone by using a NeoBase 2 Non-Derivatized MSMS Kit (Revvity). The FIA-MS/MS system consisted of the Renata DX Screening System (Waters Corporation, Milford, MA, USA). The system was operated in positive electrospray ionization mode by multiple reaction monitoring (MRM) acquisition. A total of 10 μL was injected into the ion source, and the run time was 1.1 min, injection-to-injection. The data were processed by MassLynx V4.2 and IonLynx V4.2 Software (Waters Corporation, Milford, MA, USA).

For 2-TT analysis, two DBS disks (equivalent to approximately 6–6.4 μL whole blood) were extracted for the determination of mma, mca, and hcy by LC-MS/MS, as previously detailed [5]. The LC-MS/MS system consisted of an ACQUITY UPLC I-Class/Xevo TQD IVD (Waters Corporation, Milford, MA, USA). The system was operated in positive electrospray ionization mode using TargetLynx XS V4.2 software (Waters Corporation, Milford, MA, USA). A total of 5 μL was injected into the ion source, and the run time was 9 min, using an ACQUITY UPLC BEH C18 2.1 × 50 mm column with an ACQUITY UPLC BEG C18 VanGuard pre-column. The flow gradient profile consisted of the following steps: increase from initial conditions 95% A (H_2_O + 0.1% formic acid) and 5% B (ACN + 0.1% formic acid) to 90% B within 5 min, holding for 1 min before returning to initial conditions. The flow rate was 0.5 mL/min, and the column was maintained at 40 °C.

**Figure 1:**
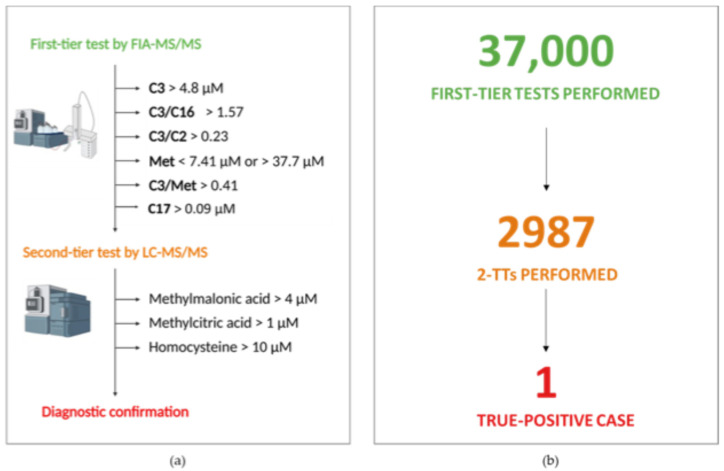
(**a**) Representation of the NBS laboratory protocol for propionate metabolism-related diseases, homocystinuria, and remethylation disorders, with the original cut-off values for first- and second-level tests. (**b**) Summary of the number of NBS and 2-TT tests performed, as well as confirmed diagnoses. Image Created in Bio-render. Claudia Rossi. (2025) https://BioRender.com.

Of the 37,000 consecutive samples analyzed from February 2019 to June 2023, 2987 (8.07%) showed an altered propionate metabolic profile at the first-tier test, considering the propionyl-carnitine (C3), methionine (Met), C3/acetyl-carnitine (C3/C2), C3/palmitoyl-carnitine (C3/C16), and C3/Met ratios as primary biomarkers. After 2987 2-TTs, 34 (1.13%) newborns had a positive result with a recall rate of 0.092% and a specificity of 99.91%. A flowchart of the laboratory protocol for NBS of diseases related to propionate metabolism, homocystinuria, and remethylation disorders, with the original cut-offs for first- and second-tier testing, and the number of NBS tests, 2-TTs, and confirmed diagnoses are summarized in Figure 1.

### 2.2. Creation of Layered Cut-Offs

For all the 37,000 samples analyzed at the first screening, customized layered cut-offs based on selected neonatal variables were calculated using Wallac Cut-Off Analyzer Version 2018 software (Revvity, Turku, Finland, OY). Layered cut-offs were calculated at the 99.5th percentile for C3, C3/C2, C3/C16, Met, and C3/Met based on BW, GA, and age at sampling. In our NBS laboratory, pre-term and/or low-BW newborns require additional DBS samples collected at 14 and 28 days of life. Similarly, if the baby requires blood transfusion or total parenteral nutrition (TPN), a DBS sample is collected before the procedures, 72 h after the end of the TPN, and 7 and 28 days after transfusion. Retrospectively, a receiver–operating characteristic (ROC) analysis was performed by GraphPad Prism 9.0 (GraphPad software, Inc., Boston, MA, USA) using the Wilson/Brown method with a confidence interval of 95%. The sensibility, specificity, and AUC were assessed for both the original and customized layered cut-offs calculated in the neonatal sub-population with at least 500 cases. The newly defined layered cut-offs were retrospectively applied in the evaluation of NBS propionate metabolic profiles. In addition to the NBS samples of our regional population (Abruzzo), we also retrospectively evaluated samples of true-positive newborns identified at the NBS Centres of Puglia and Liguria as an independent and external cohort.

### 2.3. Statistical Analysis

Univariate statistical analysis by the *t*-test and ANOVA was performed using GraphPad Prism (GraphPad software, Inc., Boston, MA, USA) for the neonatal samples (n = 2987) that required 2-TTs to evaluate possible differences in the levels of propionate-related biomarkers (C3, Met, C3/C16, C3/C2, and C3/Met) according to several selected variables reported in the DBS collection cards: gender (female or male), blood transfusion, gestational age (<37 or ≥37 weeks), age at sampling (<48 h or ≥48 h), maternal hypothyroidism, weight (<2 kg or ≥2 kg), mode of delivery (C-section or vaginal delivery), jaundice, maternal and/or infant cortisone therapy, neonatal antibiotic therapy, meconium disorders, and nutrition (breastfeeding, TPN, artificial nutrition, mixed nutrition). The distribution of the levels of propionate-related biomarkers in these newborns was used to define alarm thresholds, set at the 99.5th percentile for each of the selected variables, and at the 1.0th percentile for the low cut-off of Met, as listed above. The aforementioned “alarm threshold” should not be confused with the cut-off because this parameter does not determine whether the test is positive; however, it helps the laboratory to recognize potentially serious cases that need to be processed urgently.

## 3. Results

### 3.1. Setting a Layered Cut-Off According to Neonatal Variables

Using Wallac Cut-Off Analyzer software, we divided all the newborns based on the three selected variables. According to BW (<2 kg–≥2 kg), GA (full-term: >37 weeks; pre-term: <37 weeks), and age at sample collection (<48 h–>48 h), cut-off values were calculated for each possible combination. Table 1 shows the cut-offs calculated at the 99.5th percentile for C3, C3/C2, C3/C16, Met, and C3/Met according to the age at sampling up to 72 h. A low cut-off was calculated at 1.0^st^ percentile for Met.

Supplementary Table S1 shows the cut-off values calculated using the 99.5th (high cut-off) and the 1.0st (low cut-off) percentile for the propionate-related primary biomarkers for the newborns with age at collection > 72 h. These last infants were in follow-up according to special protocols, such as prematurity, blood transfusion, and drug treatments. The only true-positive case, an MMA with homocystinuria, as well as all the six cases of maternal vitamin B12 deficiency, previously found at NBS with the original cut-off, would have been successfully identified through the retrospective application of the layered cut-offs on the 37,000 newborns, as detailed in Table 2 and in Supplementary Table S2.

In particular, the positive case and the B12 deficiencies were confirmed by confirmatory analyses, including the quantification of urinary organic acids, plasma acylcarnitines and amino acids, and the assessment of both neonatal and maternal vitamin B12 status. Moreover, in the MMA-positive case, NGS sequencing confirmed mutations in the *MMACHC* gene.

Even if identified through the NBS workflow, vitamin B12 deficiencies are not included in the NBS panel, in agreement with the Italian law 167/2016 for expanded NBS. However, our policy is to promptly inform the referring clinicians of the possible deficiency for confirmation.

Receiver–operating characteristic (ROC) curves were used to discriminate the cases requiring 2-TTs from the negative cases according to either the original or customized layered cut-offs. Table S3 shows the specificity and sensitivity percentage values, as well as the area under the curve (AUC), for each analyte based on the stratification conditions that were associated with at least 500 cases, thus empowering data robustness. For most analytes, the customized layered cut-offs are associated with higher specificity values than the original cut-offs, while the sensitivity and AUC values are shared.

In addition, with the aim of ensuring the robustness and clinical applicability of the customized layered cut-offs established for all propionate-related biomarkers based on BW, GA, and age at sampling, we evaluated the performance of the proposed model using a larger dataset with more confirmed positive cases [18]. Given the limited number of the Abruzzo neonatal population, i.e., about 8000 newborns per year, the expected incidence of the diseases evaluated, and the only positive case of MMA with homocystinuria that we identified in these years, this further retrospective evaluation was essential for a more comprehensive validation of the refined layered cut-offs. In collaboration with the Centres for Neonatal Screening of Puglia and Liguria regions, we obtained and analyzed another set of positive cases (n = 9). The metabolic profile of these samples was correctly interpreted by applying the layered cut-offs. The details of the metabolic alterations detected in these additional nine neonatal cases when applying the customized layered cut-offs are summarized in Supplementary Table S4.

### 3.2. Descriptive Statistics

Among the 37,000 newborns that were retrospectively reviewed, 2987 (about 8% of the original neonatal population) required a 2-TT due to alterations of propionate-related primary biomarkers at the first-tier test. In our cohort, 49.6% of the subjects were females and 50.4% were males; 30.6% of the subjects were born pre-term, while 69.4% were delivered after 37 gestational weeks. Overall, 6.3% of the newborns underwent DBS collection before 48 h, whilst 93.7% were sampled as normally recommended, between 48 h and 72 h. Of the studied newborns, 12.5% had a low BW (less than 2 kg), whereas 87.5% were born weighing more than 2 kg. We found that 9.4% of the newborns had mild infant jaundice, which resolved spontaneously over time. Regarding mode of delivery, 40.7% of the newborns were born by caesarean section (C-section), while 59.3% were delivered naturally. Only 0.3% of the newborns suffered from meconium ileus. Regarding neonatal treatments, 3.1% of the babies received blood transfusions, 26.3% were treated with antibiotics, and 6.3% underwent cortisone therapy. With reference to maternal variables, 12.1% of the newborns were from hypothyroid mothers, whilst 0.5% had mothers under cortisone therapy at childbirth. As depicted in Supplementary Figure S1, most of the newborns were fed by mixed nutrition (32.14%), followed by breastfeeding (31.07%), artificial milk (10.21%), and TPN (9.91%). Some DBS cards lacked nutritional information (16.67%) and were therefore not considered in this study. Supplementary Table S5 reports a set of descriptive statistics for those samples that underwent 2-TTs in terms of either neonatal or maternal parameters.

### 3.3. Insights into the Influence of Neonatal and Maternal Variables on Propionate-Related Biomarker Levels

**Figure 2:**
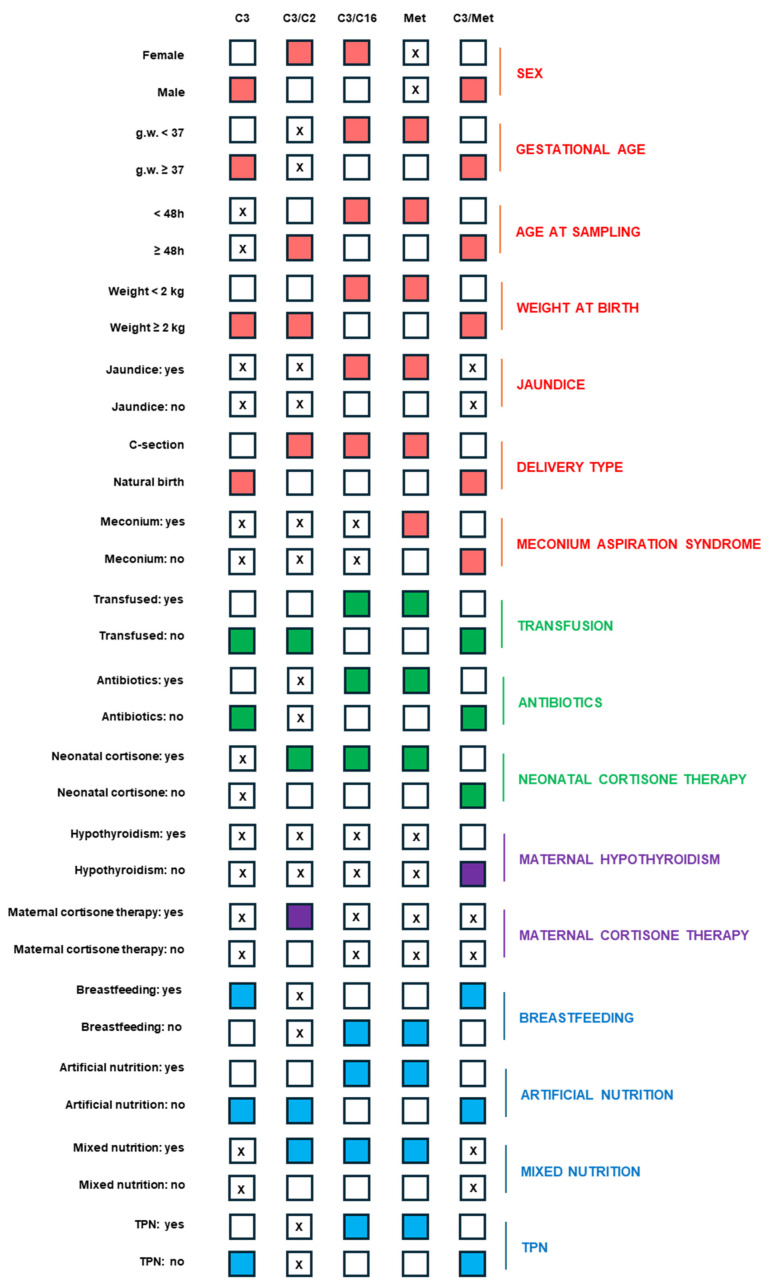
Trends in the primary biomarkers used for homocystinuria and propionate-related organic acidemia 2-TTs (C3, C3/C2, C3/C16, Met, C3/Met) based on the variables under investigation. For each category, the colored squares (red for neonatal conditions, green for neonatal treatments, violet for maternal variables, blue for nutrition) highlight when the measured levels were significantly higher for each comparison. The squares with an X indicate a lack of statistical significance in the comparison.

Figure 2 displays the neonatal and maternal variables that were evaluated to highlight trends in the propionate-related biomarkers found to be altered at the first-level test.

Sex significantly affected the expression of all propionate-related primary biomarkers, except for Met (*p*-value: 0.4256). The females had higher C3/C2 (*p*-value < 0.0001) and C3/C16 (*p*-value: 0.0367) ratios than the males, whilst the latter displayed higher C3 (*p*-value: 0.0076) and C3/Met (*p*-value < 0.0001) values.

Gestational age appeared to be related to the entire propionate-related panel, except for the C3/C2 ratio (*p*-value: 0.1632). The pre-term babies showed higher C3/C16 (*p*-value < 0.0001) and Met (*p*-value < 0.0001) levels than the full-term ones, while the latter had increased C3 (*p*-value < 0.0001) and C3/Met (*p*-value < 0.0001) values. Similarly, the newborns with age at collection < 48 h had higher C3/C16 (*p*-value < 0.0001) and Met (*p*-value < 0.0001) levels compared to the babies with age at sampling > 48 h, who showed higher C3/C2 (*p*-value: 0.0379) and C3/Met (*p*-value < 0.0001) values, with no statistically significant differences for C3 (*p*-value: 0.7404). A similar trend was observed when assessing the propionate-related biomarker levels in the newborns according to their birth weight. The babies with low birth weight showed higher values of C3/C16 (*p*-value < 0.0001) and Met (*p*-value < 0.0001), whereas those with normal birth weight had higher C3 (*p*-value < 0.0001), C3/C2 (*p*-value < 0.0001), and C3/Met (*p*-value < 0.0001) levels. Despite the small number of recorded jaundice cases, such a condition seems to partially affect the propionate-related panel, as shown by significant C3/C16 (*p*-value < 0.0001) and Met (*p*-value < 0.0001) increases in the newborns with jaundice at birth compared to the healthy subjects, while having comparable C3 (*p*-value: 0.7494), C3/C2 (*p*-value: 0.0875), and C3/Met (*p*-value: 0.1105) levels. Regarding the type of childbirth, higher C3 (*p*-value < 0.0001) and C3/Met (*p*-value < 0.0001) levels were found in the newborns with vaginal birth, while the values of C3/C2 (*p*-value: 0.0182), C3/C16 (*p*-value < 0.0001), and Met (*p*-value < 0.0001) were higher in those delivered by C-section. Meconium ileus could increase Met (*p*-value < 0.0001) and decrease C3/Met (*p*-value: 0.0028) levels in the babies experiencing it at birth, with no statistically significant differences for C3 (*p*-value: 0.8246), C3/C2 (*p*-value: 0.4383), and C3/C16 (*p*-value: 0.3218) values compared to the healthy newborns.

Regarding neonatal treatments, higher C3/C16 (*p*-value < 0.0001) and Met (*p*-value < 0.0001) levels were found in the transfused compared to the non-transfused babies, the latter showing elevated levels of C3 (*p*-value < 0.0001), C3/C2 (*p*-value < 0.0001), and C3/Met (*p*-value < 0.0001). Likewise, the newborns who were treated with antibiotics showed higher C3/C16 (*p*-value < 0.0001) and Met (*p*-value < 0.0001) values than those who were not; they had higher C3 (*p*-value < 0.0001) and C3/Met (*p*-value < 0.0001) levels, while their C3/C2 levels were not significantly different (*p*-value: 0.4712). With a similar trend in the blood transfusion and antibiotics treatments, the C3/C16 (*p*-value < 0.0001), Met (*p*-value < 0.0001), and C3/C2 (*p*-value: 0.0154) values were higher in the newborns who received cortisone therapy; the C3/Met (*p*-value < 0.0001) levels were lower, while the C3 (*p*-value: 0.4287) concentrations were comparable between those who were cortisone-treated and those who were not.

Concerning the maternal variables, the C3/Met (*p*-value: 0.0318) ratio was lower in the newborns whose mothers had thyroid disease, but no other statistically significant differences were observed for C3 (*p*-value: 0.1588), C3/C2 (*p*-value: 0.4211), C3/C16 (*p*-value: 0.6767), and Met (*p*-value: 0.5342). The newborns whose mothers received antenatal corticosteroids showed a higher C3/C2 (*p*-value: 0.0203) ratio than those with untreated mothers, whereas the C3 (*p*-value: 0.6566), C3/C16 (*p*-value: 0.0965), Met (*p*-value: 0.2200), and C3/Met (*p*-value: 0.4098) levels had no significant differences.

Nutrition can be considered as another key modulator of propionate-related biomarker levels. As a matter of fact, the breastfed infants had higher levels of C3 (*p*-value < 0.0001) and C3/Met (*p*-value < 0.0001) compared to the non-breastfed babies, who had higher Met (*p*-value < 0.0001) and C3/C16 (*p*-value < 0.0001) values, whilst no statistically significant differences were observed for the C3/C2 (*p*-value: 0.9996) ratio. Artificial nutrition produced higher C3/C16 (*p*-value < 0.0001) and Met (*p*-value < 0.0001) values compared to the newborns who did not receive formula milk and had higher C3 (*p*-value: 0.0125), C3/C2 (*p*-value: 0.0076), and C3/Met (*p*-value < 0.0001) levels. The newborns fed with mixed nutrition (i.e., combining formula milk with breastfeeding) showed higher C3/C2 (*p*-value: 0.0172), C3/C16 (*p*-value: 0.0030), and Met (*p*-value < 0.0001) levels compared to those who received other types of nutrition; however, no statistically significant differences for the C3 (*p*-value: 0.5687) and C3/Met (*p*-value: 0.0658) values were found. Lastly, the infants receiving TPN showed higher levels of C3/C16 (*p*-value < 0.0001) and Met (*p*-value < 0.0001) compared to those not receiving TPN, who instead had higher C3 (*p*-value < 0.0001), C3/C16 (*p*-value < 0.0001), and C3/Met (*p*-value < 0.0001) values, whereas no significant difference was found for the C3/C2 ratio (*p*-value: 0.5198).

All statistically significant differences described herein are reported in Supplementary Figures S2–S5 for the neonatal conditions, newborn treatments, maternal variables, and infant nutrition, respectively.

### 3.4. Alarm Thresholds for Propionate-Related Biomarkers Based on Neonatal and Maternal Variables to Define the Degree of Urgency for 2-TT Execution

In addition to calculating layered cut-offs, we also created alarm thresholds, taking into account the impact of neonatal and maternal variables on the propionate-related biomarkers. In more detail, alarm thresholds were calculated as the 99.5th percentile for the biomarkers of interest and also as the 1.0st percentile for the Met values on the 2987 newborns who underwent 2-TTs. The alarm thresholds based on the selected variables—such as newborn sex, gestational weeks, age at sampling, birth weight, type of delivery, jaundice, meconium, neonatal treatments, nutrition, and maternal factors—are reported in Table 3. The alarm thresholds are not intended to decide whether or not to execute the second-tier test, but they are designed to establish the degree of priority/urgency to perform a 2-TT only after the application of the layered cut-offs.

### 3.5. Post-Analytic Evaluation

**Figure 3:**
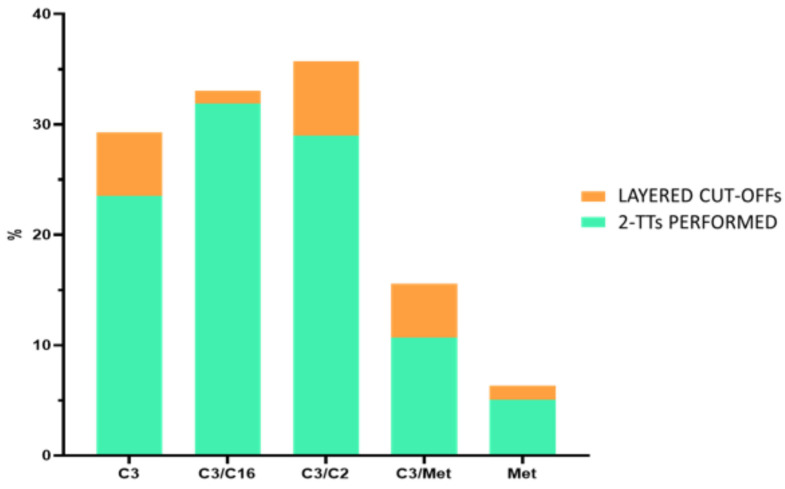
Comparison between 2-TTs performed and those that would have been performed if we had used the layered cut-offs.

Figure 3 compares the percentage of 2-TTs we executed according to the original cut-offs with those we would have performed retrospectively applying the customized layered cut-off levels (calculated by Wallac Cut-Off Analyzer software). Based on the original cut-off (C3 < 4.8 μM), C3 was found to be altered in 23% of the samples that underwent a 2-TT: the application of the layered cut-off values would have led to fewer 2-TTs (5.7%). Accordingly, the alteration of C3/C16 (cut-off C3/C16 < 1.57) at the first-tier level imposed 2-TT execution on 31.88% of the samples, while only 1.1% would have required a 2-TT applying the customized cut-offs. Regarding C3/C2 alterations (cut-off C3/C2 < 0.23), we would have found that 6.7% of the samples required a 2-TT (29%) using the layered cut-off levels. The altered C3/Met ratios (cut-off C3/Met < 0.41) were responsible for the execution of 2-TTs on 10.67% of the cases; however, the use of the customized cut-offs would have led to fewer analyses (4.9%). Likewise, 2-TTs were performed on 5.03% of the samples following Met level alterations (n.v. Met = 7.41–37.7 μM) at the first-tier test, while only 1.2% would have been analyzed by a 2-TT when applying the layered cut-offs.

Interestingly, only 1.47% of the samples (n = 544) from February 2019 to June 2023 would have required a 2-TT retrospectively using a layered cut-off.

## 4. Discussion

The introduction of MS/MS in NBS programs and the use of 2-TTs favored rapid and precise detection of IEMs, significantly reducing the number of false positives identified at the first level [2,11]. However, the number of negative tests at the 2-TT is still very high, and not all newborns positive at the 2-TT are affected by the targeted disorder. For this reason, we focused on all newborns who tested positive for propionyl carnitine metabolism screened in the NBS laboratory of the Abruzzo region from February 2019 to June 2023. Analyzing the data obtained from around 37,000 newborns, 2987 (8.07%) of them resulted positive at the first-tier test and required a 2-TT for alterations in C3, C3/Met, C3/C2, Met, and C3/C16. Of them, 34 (1.13%) newborns tested positive after the 2-TT. Only seven infants required further confirmatory testing: six newborns were defined as having maternal vitamin B12 deficiency, and one finally received a diagnosis of MMA with homocystinuria.

Given this high number of 2-TTs despite very few positive cases, we tried to increase the specificity of the primary markers by creating layered cut-offs according to GA, BW, and age at sampling in our neonatal population. We clearly demonstrated that by applying the cut-offs adjusted for GA, BW, and age at DBS collection, the number of newborns requiring a 2-TT would have dropped from 8.07% to only 1.47%. In this scenario, we suggest refining the NBS strategy by applying layered cut-off values that take into account factors such as BW, GA, and age at DBS collection. This allows for increasing the specificity of biomarkers of disorders of propionate metabolism, homocystinuria, and remethylation disorders, thus reducing the laboratory’s workload.

Applying the layered cut-offs to all the 37,000 newborns screened, we would have correctly identified the single case of MMA with homocystinuria and the six cases of maternal vitamin B12 deficiencies, which were previously diagnosed using our original cut-offs. Yet, to further support the clinical applicability and confirm the potential of our model, we retrospectively evaluated nine additional positive cases from an extra-regional neonatal population using the new layered cut-offs [18]. As shown in Table S4, all nine cases were classified as positives, thus strengthening the potential of the proposed layered cut-offs.

It is well known that external factors can influence analytical data; therefore, we also investigated if other neonatal and maternal variables, such as weight, GA, type of nutrition, sex, jaundice, meconium, maternal hypothyroidism, type of delivery, and pharmacological treatments, could influence the propionate-related biomarker levels.

A first observation was that infants requiring a 2-TT for higher Met levels at NBS are often formula fed. This is in keeping with knowledge that formula milk contains a greater quantity of Met compared to breast milk, potentially resulting in hypermethioninemia caused by excessive ingestion of Met [19]. Similarly, newborns receiving TPN had higher concentrations of Met as a consequence of the exogenous administration of sulfur-containing amino acids, including methionine, cysteine, and cystine [20]. As expected, high concentrations of Met found in pre-term newborns result not only from formula feeding or TPN, but also from their immature enzymatic system, which is unable to properly metabolize this essential amino acid [21]. Indeed, the proposed customized cut-offs show that Met levels are much higher in low-BW and pre-term infants (Table 1 and Table 3). For cases of prematurity, low BW, transfusion, and TPN, our laboratory follows special protocols that require the monitoring of Met levels at different timepoints.

Our data confirm the idea that the phenotype is not only influenced by the genome but also by environmental factors and clinical variables; indeed, we show that these factors can impact the profile of the NBS biomarker panel. Furthermore, we believe that this retrospective approach could also be useful for other conditions besides propionyl carnitine-related disorders, in particular by examining those markers that generate a high number of false positives in screening tests.

## 5. Conclusions

Our results clearly demonstrate that the application of customized layered cut-off values allows for the limitation of unnecessary 2-TTs, altogether improving analytical performance while retaining specificity and sensitivity.

Furthermore, in an effort to better prioritize the laboratory workload, we also calculated alarm thresholds to define the urgency in the execution of 2-TTs.

However, we would like to stress the fact that alarm thresholds are not intended to be used as cut-offs or to decide whether or not to execute 2-TTs. They are only intended to anticipate urgent 2-TTs, while non-urgent 2-TTs are performed within the response time schedule, as defined by NBS protocols.

We have not yet started using customized layered cut-offs and alarm thresholds in the NBS routine in our lab since we want to confirm these results on a larger population or a different dataset. However, we believe that this represents an example of a good working model of precision and personalized medicine that will lead, in the near future, to the development of new approaches that will improve the diagnostic process and favor an optimization of costs and laboratory workload, preventing unnecessary recall and referral of newborns without missing true positives.

## Figures and Tables

**Table 1:** Cut-offs calculated for all propionate-related biomarkers based on weight, age at sampling, and gestational weeks for newborns with an age at collection up to 72 h. Cut-offs were calculated using the 99.5th (high cut-off) and 1.0st percentile (low cut-off).

Panel	C3	C3/C2	C3/C16	Met	C3/Met
**Weight ≥ 2 kg, GA > 37, age at sampling > 48 h** (n = 29,595)	6.10	0.28	1.83	7.36–30.99	0.47
**Weight ≥ 2 kg, GA > 37, age at sampling < 48 h** (n = 1674)	7.75	0.32	2.36	6.8–43.96	0.50
**Weight ≥ 2 kg, GA < 37, age at sampling < 48 h** (n = 290)	9.30	0.48	3.89	8.47–69.58	0.64
**Weight ≥ 2 kg, GA < 37, age at sampling > 48 h** (n = 1453)	7.48	0.41	2.79	8.47–44.73	0.64
**Weight < 2 kg, GA < 37, Age at sampling < 48 h** (n = 397)	7.30	0.36	3.32	7.10–128.93	0.65
**Weight < 2 kg, GA < 37, age at sampling > 48 h** (n = 504)	7.88	0.44	5.49	7.25–108.65	0.64
**Weight < 2 kg, GA > 37, age at sampling < 48 h** (n = 15)	3.85	0.18	1.32	5.24–28.14	0.33
**Weight < 2 kg, GA > 37, age at sampling > 48 h** (n = 70)	4.64	0.24	2.80	9.41–41.05	0.29

**Table 2:** Results of propionate-related primary biomarkers measured at MS/MS NBS for the confirmed case of MMA with homocystinuria, when retrospectively applying customized layered cut-offs.

Sample Code	C3 (Cut-Off < 6.10 μM)	C3/C2 (Cut-Off < 0.28)	C3/C16 (Cut-Off < 1.83)	Met (n.v. 7.36–30.99 μM)	C3/Met (Cut-Off < 0.47)	Confirmed Diagnosis
Sample 001	7.75	0.71	5.88	3.37	2.3	MMA (CbIC)

**Table 3:** Alarm thresholds calculated for all propionate-related biomarkers based on neonatal conditions and treatment, nutrition, and maternal variables.

	C3	C3/C2	C3/C16	Met	C3/Met	
Female	8.97	0.50	5.11	9.71–64.06	0.77	**NEONATAL ** **CONDITIONS**
Male	10.13	0.44	5.33	8.05–65.14	0.83	
GA < 37	9.25	0.52	5.47	10.8–93.77	0.68	
Age at sampling < 48 h	8.67	0.45	5.50	8.84–88.42	0.63	
Weight < 2 kg	8.20	0.71	7.94	9.74–161.68	0.61	
Weight ≥ 2 kg	9.77	0.45	4.19	8.87–51.39	0.79	
Natural birth	9.63	0.44	5.05	8.34–62.39	0.79	
C-section	10.36	0.46	5.78	10.2–67.63	0.76	
Jaundice	11.26	0.39	15.25	10.3–83.8	0.92	
Meconium	7.78	0.29	1.98	9.23–59.81	0.76	
Transfusion	6.35	0.48	6.48	9.10–91.28	0.41	**NEONATAL ** **TREATMENTS**
Antibiotics	10.41	0.44	7.71	11.0–94.33	0.77	
Neonatal cortisone	8.58	0.86	5.94	10.32–90.47	0.52	
Breastfeeding	9.16	0.45	3.78	6.92–48.23	0.78	**NUTRITION**
Artificial nutrition	8.34	0.84	8.10	11.38–119.66	0.79	
Mixed nutrition	10.40	0.45	7.73	9.85–50.94	0.77	
TPN	9.54	0.44	5.43	12.9–93.76	0.53	
Maternal cortisone	5.95	0.32	2.44	11.13–38.63	0.45	**MATERNAL ** **VARIABLES**
Hypothyroidism	9.79	0.40	4.19	8.77–58.7	0.81	

## Data Availability

The data presented in this study are available on request from the corresponding author due to privacy and ethical reasons.

## Supplementary Materials

The following supporting information can be downloaded at https://www.mdpi.com/article/10.3390/metabo15050308/s1: Table S1: Cut-offs calculated for all propionate-related biomarkers based on weight, age at sampling, and gestational weeks for newborns with an age at sampling between 73 and 672 h of life. Cut-offs calculated at the 99.5th percentile. Table S2: Propionate-related primary biomarkers as measured at MS/MS NBS for the six confirmed cases of vitamin B12 deficiency, retrospectively applying the customized layered cut-off. Table S3: Specificity, sensitivity, and AUC based on the original cut-off and customized layered cut-offs are listed for C3, C3/C2, C3/C16, Met, and C3/Met. Table S4: Propionate-related primary biomarkers as measured at MS/MS NBS for the eight additional confirmed cases (external evaluation dataset) of diseases related to propionate metabolism, homocystinuria, and remethylation disorders, retrospectively applying the customized layered cut-off. Table S5: Descriptive statistics of the newborns under investigation in terms of neonatal conditions, treatments, and maternal variables. Figure S1: Pie chart showing the percentage of newborns in each feeding category. Figure S2: Dot plots of propionate-related biomarkers based on neonatal conditions (i.e., sex, gestational age, age at sampling, weight at birth, jaundice, type of delivery, and meconium pathology). Figure S3: Dot plots of propionate-related biomarkers based on neonatal treatments (i.e., blood transfusion, antibiotics, and cortisone therapy). Figure S4: Dot plots of propionate-related biomarkers based on maternal variables (i.e., hypothyroidism, antibiotics, and cortisone therapy). Figure S5: Dot plots of propionate-related biomarkers based on neonatal nutrition (i.e., breastfeeding, artificial, mixed, and TPN).
