# Supplementary material for: Propionyl Carnitine Metabolic Profile: Optimizing the Newborn Screening Strategy Through Customized Cut-Offs
Source: Metabolites. 2025 May 6;15(5):308. doi: 10.3390/metabo15050308 (PMC12112895; doi:10.3390/metabo15050308)

Supplementary Materials

Article

Propionyl Carnitine Metabolic Profile: Optimizing the Newborn Screening Strategy Through Customized Cut-Offs

Maria Lucia Tommolini [1,2,†], Maria Concetta Cufaro [1,2,†], Silvia Valentinuzzi [1,3,†], Ilaria Cicalini [1,2], Mirco Zucchelli [1,2], Alberto Frisco [1,4], Simonetta Simonetti [5], Michela Perrone Donnorso [6,7], Sara Moccia [2], Ines Bucci [1,8], Maurizio Aricò [9], Vincenzo De Laurenzi [1,2], Luca Federici [1,2], Damiana Pieragostino [1,2] and Claudia Rossi [1,3,*]

1 Center for Advanced Studies and Technology (CAST), "G. d'Annunzio" University of Chieti-Pescara, 66100 Chieti, Italy; maria.tommolini@unich.it (M.L.T.); maria.cufaro@unich.it (M.C.C.); silvia.valentinuzzi@unich.it (S.V.); ilaria.cicalini@unich.it (I.C.); mirco.zucchelli@unich.it (M.Z.); alberto.frisco@unidav.it (A.F.); ines.bucci@unich.it (I.B.); vincenzo.delaurenzi@unich.it (V.D.L.); luca.federici@unich.it (L.F.); damiana.pieragostino@unich.it (D.P.)
2 Department of Innovative Technologies in Medicine and Dentistry, "G. d'Annunzio" University of Chieti-Pescara, 66100 Chieti, Italy; sara.moccia@unich.it
3 Department of Science, "G. d'Annunzio" University of Chieti-Pescara, 66100 Chieti, Italy
4 Department of Human, Legal and Economic Sciences, Telematic University of "Leonardo Da Vinci", 66010 Torrevecchia Teatina, Italy
5 Regional Centre for Neonatal Screening, Department of Clinical Pathology and Neonatal Screening, Children's Hospital "Giovanni XXIII", Azienda Ospedaliero-Universitaria Consorziale, 70126 Bari, Italy; simonetta.simonetti@policlinico.ba.it
6 Laboratory for the Study of Inborn Errors of Metabolism, Pediatric Clinic, IRCCS Instituto Giannina Gaslini, 16147 Genoa, Italy; michelaperronedonnorso@gaslini.org
7 Department of Neuroscience, Rehabilitation, Ophthalmology, Genetics, Maternal and Child Health, University of Genoa, 16126 Genoa, Italy
8 Department of Medicine and Aging Science, "G. d'Annunzio" University of Chieti-Pescara, 66100 Chieti, Italy
9 U.O.C. Pediatrics, S. Spirito Hospital, ASL Pescara, 65124 Pescara, Italy; maurizio.arico@asl.pe.it
* Correspondence: claudia.rossi@unich.it; Tel.: +39-0871-541290
† These authors equally contributed to the work and share first authorship.

Content summary: The following supported information contains 5 supplementary tables and 3 supplementary figures as detailed. Table S1 detailed cut-offs calculated for all propionate-related biomarkers based on weight, age at sampling and gestational weeks for newborns with an age at sampling between 73 and 672 hours of life. Cut-offs calculated at the 99.5th percentile. Table S2 conteins propionate-related primary biomarkers as measure at MS/MS NBS for the six confirmed case of vitamin B12 deficiency, retrospectively applying customized layered cut-off. Table S3 listed specificty, sensitivity and AUC based on Original cut-off and Customized layered cut-off are listed for C3, C3/C2, C3/C16, Met and C3/Met. Table S**4** describes Propionate-related primary biomarkers as measure at MS/MS NBS for the eight additional confirmed case (external evaluation dataset) of diseases related to the propionate metabolism, homocystinuria and remethylation disorders, retrospectively applying customized layered cut-off. Table S**5** Descriptive statistics of newborns under investigation in term of neonatal conditions, treatments and maternal variables. Figure S1:Pie chart showing the percentage of newborns in each feeding category. Figure S2: Dot plots of propionate-related biomarkers based on neonatal conditions (i.e., sex, gestational age., age at sampling, weight at birth, jaundice, type of delivery and meconium pathology). Figure S3: dot plots of propionate-related biomarkers based on neonatal treatments (i.e., blood transfusion, antibiotics, cortisone therapy). Figure S4: dot plots of propionate-related biomarkers based on maternal variables (i.e., hypothyroidism, antibiotics, cortisone therapy). Figure S5: dot plots of propionate-related biomarkers based on neonatal nutrition (i.e., breastfeeding, artificial, mixed and TPN).

Table S1: Cut-offs calculated for all propionate-related biomarkers based on weight, age at sampling and gestational weeks for newborns with an age at sampling between 73 and 672 hours of life. Cut-offs calculated using the 99.5th (high cut-off) and 1.0th percentile (low cut-off).

Panel	C3	C3/C2	C3/C16	Met	C3/Met
**Weight ≥ 2Kg, GA > 37, Age at sampling > 72h** (n=4519)	5.71	0.29	3.14	8.47-36.43	0.36
**Weight ≥ 2Kg, GA < 37, Age at sampling > 72h** (n=1265)	5.50	0.30	4.25	8.72-40.45	0.34
**Weight < 2Kg, GA < 37, Age at sampling > 72h** (n=851)	6.05	0.30	4.42	8.61-51.94	0.38
**Weight < 2Kg, GA > 37, Age at sampling > 72h** (n=48)	4.09	0.32	1.69	8.12-46.30	0.27

Table S2. Propionate-related primary biomarkers as measure by MS/MS NBS for the six confirmed case of maternal vitamin B12 deficiency, retrospectively applying customized layered cut-off.

Sample code	C3 (cut-off < 6.10 μM)	C3/C2 (cut-off < 0.28)	C3/C16 (cut-off < 1.83)	Met (n.v. 7.36 – 30.99 μM)	C3/Met (cut-off < 0.47)	Confirmed diagnosis
**Abr001**	5.90	**0.36**	**1.98**	10.1	**0.58**	**Maternal Vit_B12 deficiency**
**Abr002**	3.85	**0.31**	1.76	**7.0**	**0.55**	**Maternal Vit B12 deficiency**
**Abr003**	5.96	**0.39**	1.45	12.7	**0.47**	**Maternal Vit B12 deficiency**
**Abr004**	**7.05**	**0.42**	**2.77**	21.4	0.33	**Maternal Vit B12 deficiency**
**Abr005**	5.99	**0.33**	1.33	**6.16**	**0.97**	**Maternal Vit B12 deficiency**
**Abr006**	**6.77**	**0.30**	1.14	9.89	**0.68**	**Maternal Vit B12 deficiency**

Table S3: Specificity, Sensitivity and AUC based on original cut-off and customized layered cut-off are listed for C3, C3/C2, C3/C16, Met and C3/Met.

**Condition 1**: weight ≥ 2Kg, GA > 37, age at sampling > 48h (N= 29595)				
**Analyte**	**Cut-off**	**Specificity**	**Sensitivity**	**AUC**
C3	Original: > 4.8 μM	98.45	100	0.9997
	Customized layered: > 6.1 μM	99.97	100	0.9997
C3/C2	Original: > 0.23	99.56	100	1
	Customized layered: > 0.28	99.85	100	1
C3/C16	Original: > 1.57	99.98	100	1
	Customized layered: > 1.83	99.98	100	1
Met	Original: < 7.41 μM	100	100	1
	Customized layered: < 7.36 μM	100	100	1
	Original: > 37.7 μM	100	100	1
	Customized layered: > 30.99 μM	100	100	1
C3/Met	Original: > 0.41	99.85	100	1
	Customized layered: > 0.47	99.85	100	1
**Condition 2**: weight ≥ 2Kg, GA > 37, age at sampling < 48h (N= 1674)				
**Analyte**	**Cut-off**	**Specificity**	**Sensitivity**	**AUC**
C3	Original: > 4.8 μM	100	100	1
	Customized layered: > 7.75 μM	100	100	1
C3/C2	Original: > 0.23	99.08	100	1
	Customized layered: > 0.32	99.88	100	1
C3/C16	Original: > 1.57	99.76	100	1
	Customized layered: > 2.36	99.94	100	1
Met	Original: < 7.41 μM	99.94	100	1
	Customized layered: < 6.8 μM	100	100	1
	Original: > 37.7 μM	99.94	100	1
	Customized layered: > 43.96 μM	99.94	100	1
C3/Met	Original: > 0.41	100	100	1
	Customized layered: > 0.50	99.76	100	1
**Condition 3**: weight ≥ 2Kg, GA < 37, age at sampling > 48h (N= 1453)				

Analyte	Cut-off	Specificity	Sensitivity	AUC
C3	Original: > 4.8 μM	100	100	1
	Customized layered: > 7.48 μM	100	100	1
C3/C2	Original: > 0.23	97.95	100	1
	Customized layered: > 0.41	99.93	100	1
C3/C16	Original: > 1.57	99.62	100	1
	Customized layered: > 2.79	100	100	1
Met	Original: < 7.41 μM	100	100	1
	Customized layered: < 8.47 μM	100	100	1
	Original: > 37.7 μM	100	100	1
	Customized layered: > 44.73 μM	100	100	1
C3/Met	Original: > 0.41	99.86	100	1
	Customized layered: > 0.64	100	100	1
**Condition 4**: weight < 2Kg, GA < 37, age at sampling > 48h (N= 504)				
**Analyte**	**Cut-off**	**Specificity**	**Sensitivity**	**AUC**
C3	Original: > 4.8 μM	100	100	1
	Customized layered: > 7.88 μM	99.8	100	1
C3/C2	Original: > 0.23	97.82	100	1
	Customized layered: > 0.44	100	100	1
C3/C16	Original: > 1.57	100	100	1
	Customized layered: > 5.49	99.8	100	1
Met	Original: < 7.41 μM	100	100	1
	Customized layered: < 7.25 μM	99.79	100	1
	Original: > 37.7 μM	100	100	1
	Customized layered: > 108.65 μM	99.79	100	1
C3/Met	Original: > 0.41	99.59	100	1
	Customized layered: > 0.64	100	100	1

Table S4. Propionate-related primary biomarkers as measure at MS/MS NBS for the nine additional confirmed case (external evaluation dataset) of diseases related to the propionate metabolism, homocystinuria and remethylation disorders, retrospectively applying customized layered cut-off.

Sample code	C3 (cut-off < 6.10 μM)	C3/C2 (cut-off < 0.28)	C3/C16 (cut-off < 1.83)	Met (n.v. 7.36 – 30.99 μM)	C3/Met (cut-off < 0.47)	Confirmed diagnosis
**ExtAbr001**	**6.29**	**0.41**	**2.33**	23,4	0.27	**MMA (CbIA)_**
**ExtAbr002**	5.68	0.25	1.34	**5.5**	**1.04**	**MMA (CbIC)_**
**ExtAbr003**	**9.22**	**0.47**	**1.91**	**5.1**	**1.81**	**MMA (CbIC)_**
**ExtAbr004**	**14.85**	**0.5**	**2.76**	8.7	1.7	**MMA (CbIC)_**
**ExtAbr005**	**8.4**	**0.32**	**1.84**	9.0	**0.93**	**MMA (CbIC)_**
**ExtAbr006**	**7.0**	**0.29**	1.26	17.9	0.39	**MMA (CbIC)_**
**ExtAbr007**	**6.99**	0.26	**1.87**	19.3	0.36	**MMA (Mut0)_**
**ExtAbr008**	1.3	0.07	0.36	**71.2**	0.02	**Homocystinuria (CBS)**
**ExtAbr009**	1.52	0.11	0.38	**4.88**	0.31	**MTHFR**

Table S5: Descriptive statistics of newborns under investigation in term of neonatal conditions, treatments and maternal variables (gestational age in weeks: GA).

N = 2987 (100%)	**Female**	**Male**	**NEONATAL CONDITIONS**
	1481 (49.6%)	1506 (50.4%)	
N = 2900 (97.1%)	**GA < 37**	**GA ≥ 37**	
	887 (30.6%)	2013 (69.4%)	
N = 2987 (100%)	**Age at sampling < 48h**	**Age at sampling > 48h**	
	189 (6.3%)	2798 (93.7%)	
N = 2864 (95.9%)	**Weight < 2Kg**	**Weight ≥ 2Kg**	
	358 (12.5%)	2506 (87.5%)	
N = 2987 (100%)	**Jaundice: yes**	**Jaundice: no**	
	281 (9.4%)	2706 (90.6%)	
N = 2987 (100%)	**C-section**	**Natural birth**	
	1217 (40.7%)	1770 (59.3%)	
N = 2987 (100%)	**Meconium: yes**	**Meconium: no**	
	8 (0.3%)	2979 (99.7%)	
N = 2987 (100%)	**Transfused: yes**	**Transfused: no**	**NEONATAL TREATMENTS**
	92 (3.1%)	2895 (96.1%)	
N = 2987 (100%)	**Antibiotics: yes**	**Antibiotics: no**	
	785 (26.3%)	2202 (73.7%)	
N = 2987 (100%)	**Cortisone therapy: yes**	**Cortisone therapy: no**	
	187 (6.3%)	2800 (93.7%)	
N = 2987 (100%)	**Cortisone therapy: yes**	**Cortisone therapy: no**	**MATERNAL VARIABLES**
	15 (0.5%)	2972 (99.5%)	
N = 2987 (100%)	**Hypothyroidism: yes**	**Hypothyroidism: no**	
	360 (12.1%)	2627 (87.9%)	

Figure S1: Pie chart showing the percentage of newborns in each feeding category.

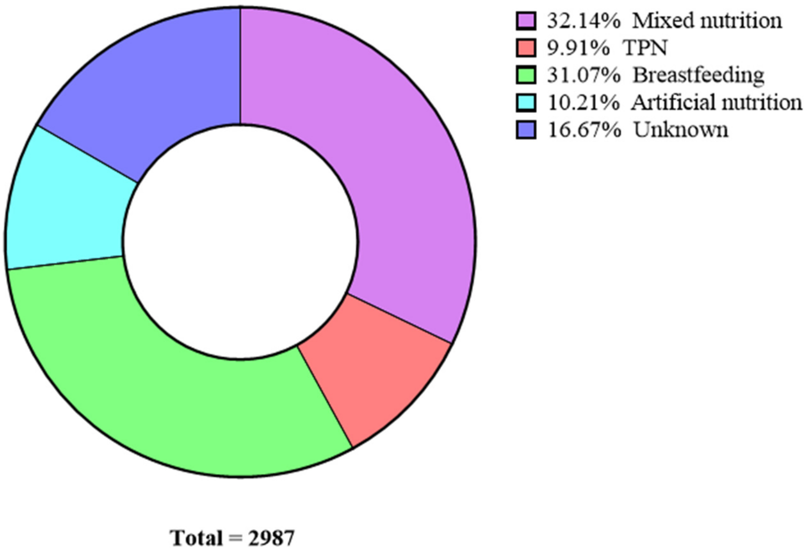

Figure S2: Dot plots of propionate-related biomarkers based on neonatal conditions (i.e., sex, gestational age., age at sampling, weight at birth, jaundice, type of delivery and meconium pathology. *: p-value < 0.05; **: p-value < 0.01; ***: p-value < 0.001; ****: p-value < 0.0001.

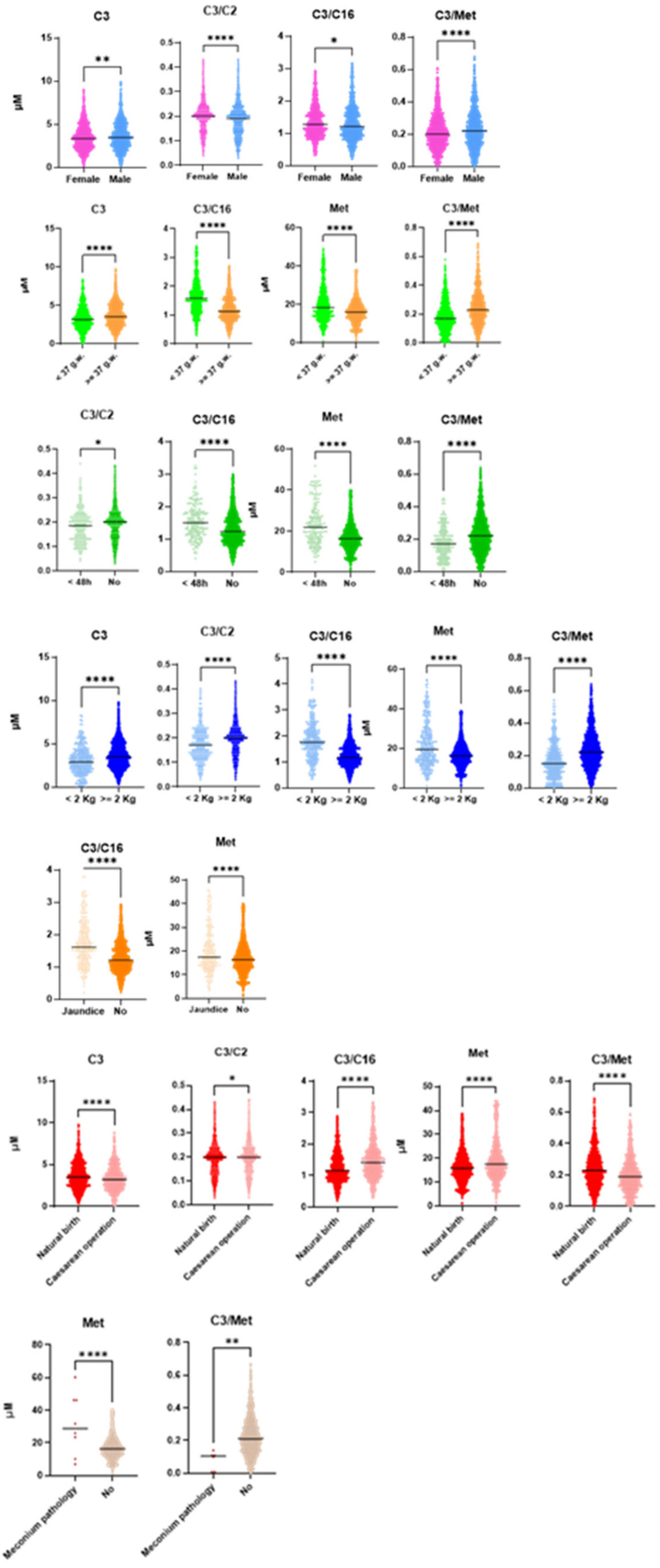

Figure S3 dot plots of propionate-related biomarkers based on neonatal treatments (i.e., blood transfusion, antibiotics, cortisone therapy). *: p-value < 0.05; **: p-value < 0.01; ***: p-value < 0.001; ****: p-value < 0.0001.

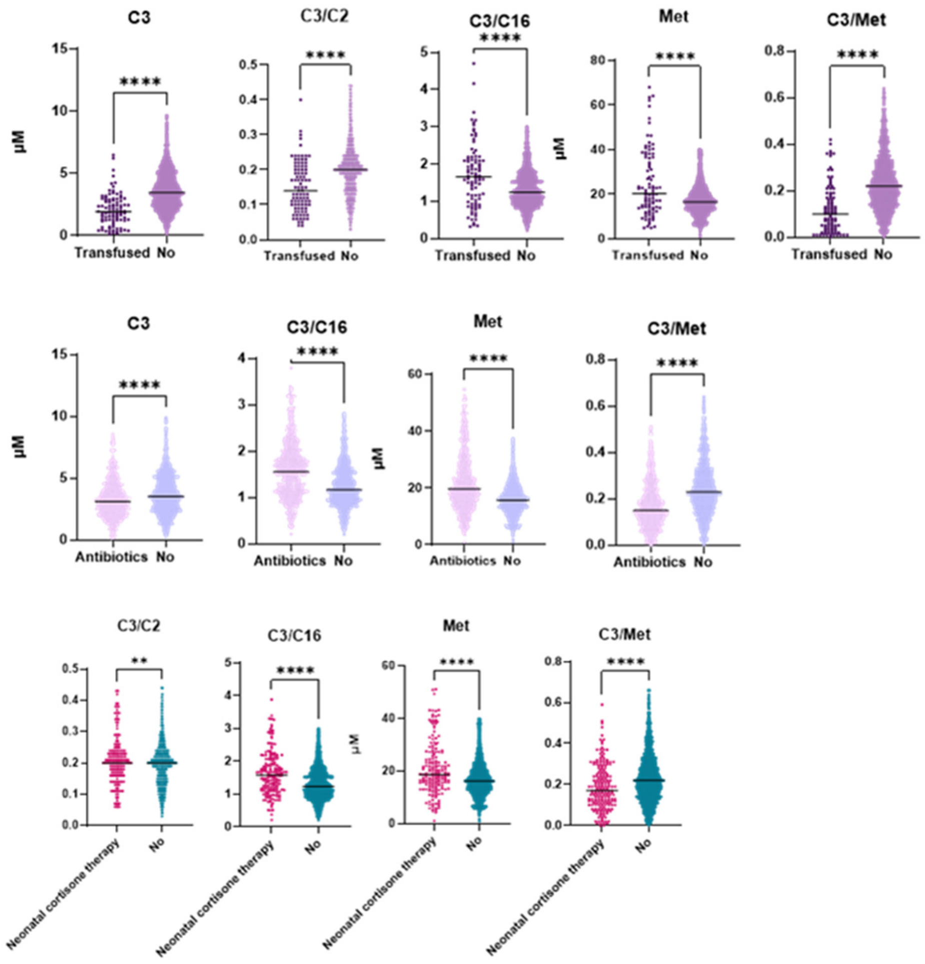

Figure S4: dot plots of propionate-related biomarkers based on maternal variables (i.e., hypothyroidism, antibiotics, cortisone therapy). *: p-value < 0.05; **: p-value < 0.01; ***: p-value < 0.001; ****: p-value < 0.0001.

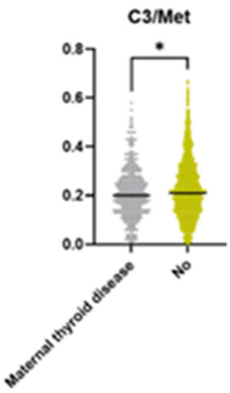

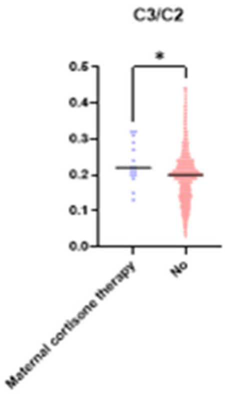

Figure S5 dot plots of propionate-related biomarkers based on neonatal nutrition (i.e., breastfeeding, artificial, mixed and TPN). *: p-value < 0.05; **: p-value < 0.01; ***: p-value < 0.001; ****: p-value < 0.0001.

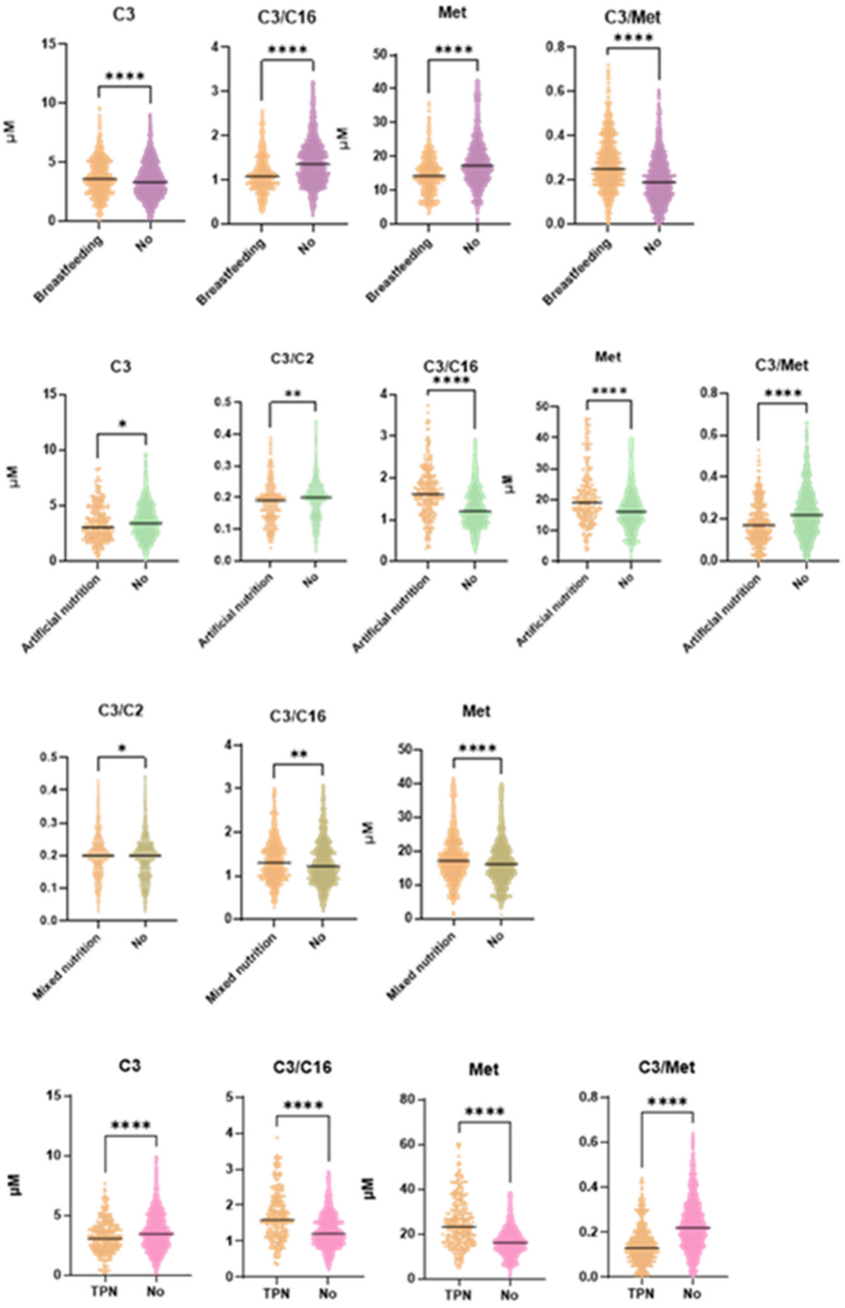

Supplement: Supplementary file 1 [file metabolites-15-00308-s001.zip › metabolites-3596859-supplementary.pdf]
